# Crystal structure of *N*,*N*′-di­decyl­pyromellitic di­imide

**DOI:** 10.1107/S2056989017006867

**Published:** 2017-05-12

**Authors:** Hansu Im, Myong Yong Choi, Cheol Joo Moon, Tae Ho Kim

**Affiliations:** a Department of Chemistry (BK21 plus) and Research Institute of Natural Sciences, Gyeongsang National University, Jinju 52828, Republic of Korea

**Keywords:** crystal structure, theoretical calculations, pyromellitic di­imide, hydrogen bond

## Abstract

The title compound, C_30_H_44_N_2_O_4_ [systematic name: 2,6-di­decyl­pyrrolo­[3,4-*f*]iso­indole-1,3,5,7(2*H*,6*H*)-tetra­one], consists of a central pyromellitic di­imide moiety with terminal decyl groups at the N-atom positions. The centre of the mol­ecule lies on a crystallographic inversion centre so the asymmetric unit contains one half-mol­ecule. The mol­ecule exhibits a rod-shaped conformation, like other similar compounds of this type, the distance between the ends of terminal decyl groups being 32.45 Å. The packing is dominated by a lamellar arrangement of the mol­ecules, which is reinforced by C—H⋯O hydrogen bonds and C—O⋯π inter­actions, forming a classic herringbone structure. The mol­ecular structure is consistent with the theoretical calculations performed by density functional theory (DFT).

## Abstract 2

The title compound comprises a central pyromellitic di­imide moiety with terminal decyl groups, with potential applications as an acaricide, insecticide and mematicide.

## Chemical context   

Previous studies have proposed that pyromellitic di­imide derivatives have potential applications in energy storage materials (Song *et al.*, 2010 ▸) and photovoltaic devices (Kanosue & Ando, 2016 ▸). Additionally, aromatic di­imides can act as organic semiconductors (Shao *et al.*, 2014 ▸). Recently, our group reported a copper(I) coordination polymer with a pyromellitic di­imide ligand, namely *N*,*N*′-bis­[2-(cyclo­hexyl­thio)­eth­yl]pyromellitic di­imide, and showed that the ligand has two conformations, *syn* and *anti*. In addition, a reversible *anti* to *syn* transition was achieved by agitating in mixed organic solvents (Kang *et al.*, 2015 ▸). In an extension of our studies of pyromellitic di­imide derivatives, we have prepared the title compound by the reaction of pyromellitic dianhydride with decyl­amine and report its crystal structure herein.
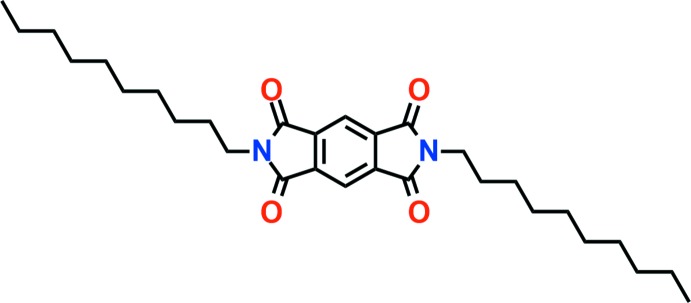


## Structural commentary   

**Figure 1:**
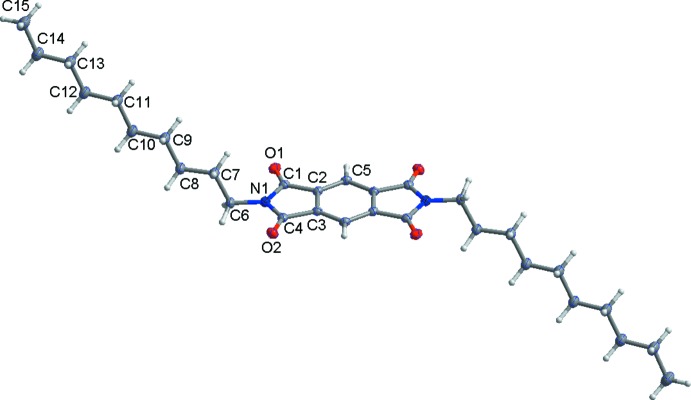
The asymmetric unit of the title compound, with displacement ellipsoids drawn at the 50% probability level. H atoms are shown as small spheres of arbitrary radius. Unlabelled atoms are generated by the symmetry operation (1 − *x*, 1 − *y*, 2 − *z*).

The title compound consists of a central pyromellitic di­imide with two terminal decyl groups (Fig. 1 ▸). The centre of the mol­ecule lies on a crystallographic inversion centre and the asymmetric unit of the title compound is composed of one half-mol­ecule. The decyl chains are inclined at an angle of 67.96° to the plane of the pyromellitic di­imide ring. The decyl chains point in opposite directions, forming a rod-shaped conformation with a distance of 32.45 Å between the carbon atoms of the terminal decyl groups.

## Supra­molecular features   

**Figure 2:**
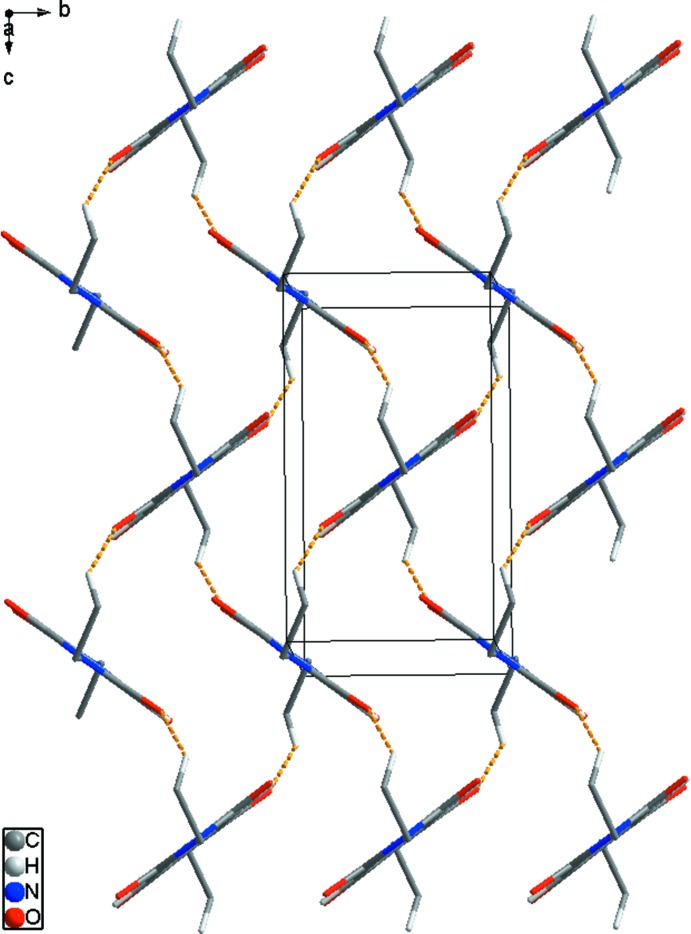
Inter­molecular C—H⋯O hydrogen bonds (yellow dashed lines) [symmetry code: (i) *x*, −*y* + 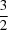, *z* − 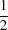] in the crystal of (I). H atoms and terminal decyl chains not involved in inter­molecular inter­actions have been omitted for clarity.

**Figure 3:**
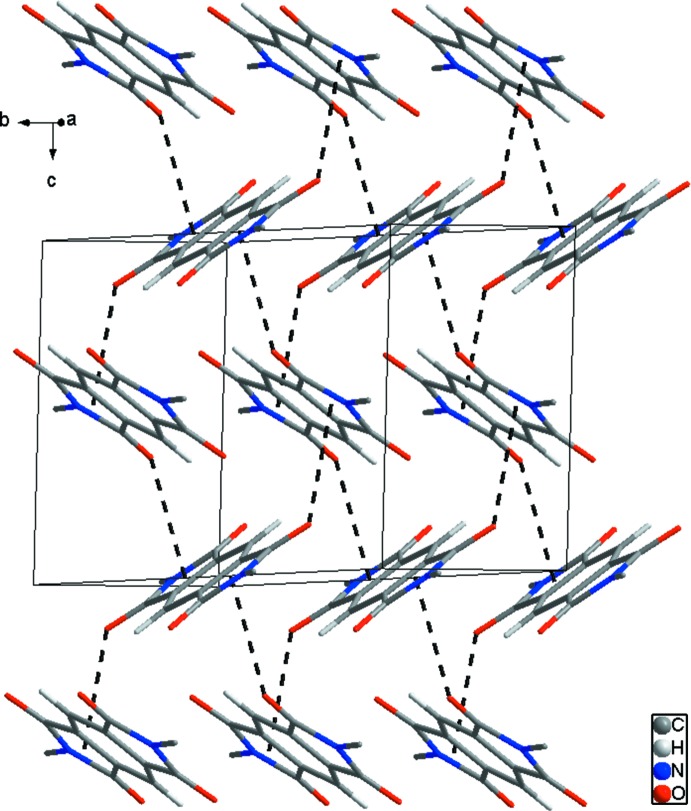
The packing diagram for (I), showing the classic herringbone structure formed by C—O⋯π inter­actions (black dashed lines). H atoms and terminal decyl chains not involved in inter­molecular inter­actions have been omitted for clarity.

**Figure 4:**
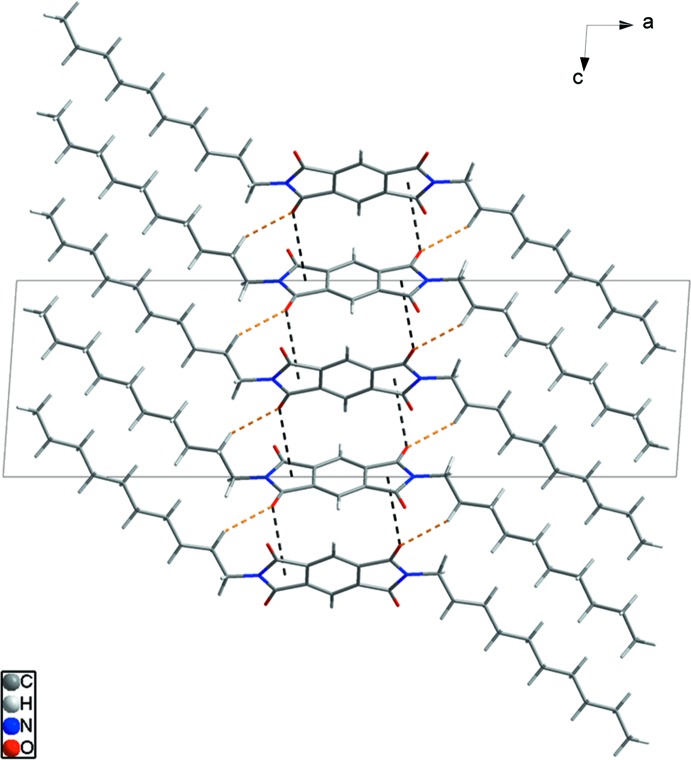
The packing for (I) showing inter­molecular hydrogen bonds (yellow dashed lines) and C—O⋯π inter­actions (black dashed lines) accepted by the same O atom.

In the crystal, C7—H7*B*⋯O2^i^ [symmetry code: (i) *x*, −*y* + 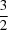, *z* − 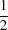; Table 1 ▸] hydrogen bonds (H⋯O = 2.57 Å) link adjacent mol­ecules (yellow dashed lines in Fig. 2 ▸). In addition,, adjacent mol­ecules are connected by C4—O2^ii^⋯*Cg*1 (*Cg*1 is the centroid of the N1/C1–C4 ring) inter­actions [O⋯π = 3.272 (1) Å; symmetry code: (ii) *x*, −*y* + 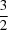, *z* + 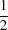], resulting in the formation of a classic herringbone structure (black dashed lines in Fig. 3 ▸). One oxygen atom accepts both hydrogen bonds and C—O⋯π inter­actions with neighboring mol­ecules, generating a two-dimensional architecture extending parallel to the *bc* plane (Fig. 4 ▸).

## Theoretical calculations   

DFT calculations have been performed to support the experimental values on the basis of the diffraction study using the *GAUSSIAN09* software package (Frisch *et al.*, 2009 ▸). Full geometry optimizations were performed using B3LYP levels of theory with a 6-311G* basis set. The optimized parameters such as bond lengths and bond angles are in excellent agreement with the experimental crystallographic data (Table 2 ▸). In particular, the theoretical value (67.07°) for the angle between the decyl chain and the plane of the pyromellitic di­imide ring is almost equal that obtained from the experimental crystallographic data (67.96°).

## Synthesis and crystallization   

A mixture of pyromellitic dianhydride (0.55g, 2.5mmol) and decyl amine (0.88 g, 5.3mmol) in toluene (10 ml) and dimethyl sulfoxide (6 ml) was heated at 453 K with stirring for 5 h. Upon cooling to room temperature, an off-white crude solid was filtered and washed with water, methanol and ether. Crystals suitable for X-ray diffraction analysis were obtained by slow evaporation of a di­chloro­methane solution of the title compound. ^1^H NMR (300 MHz, CDCl_3_): *d* = 8.27 (*s*, 2H, Ar), 3.74 (*t*, 4H, CH_2_N), 1.70 (*t*, 4H, CH_2_CH_2_N), 1.32 (*m*, 28H, CH_2_), 0.88 (*t*, 6H, CH_3_)

## Refinement   

Crystal data, data collection and structure refinement details are summarized in Table 3 ▸. All H atoms were positioned geometrically and refined using a riding model with *d*(C—H) = 0.95 Å, *U*_iso_(H) = 1.2*U*_eq_(C) for aromatic C—H, *d*(C—H) = 0.99 Å, *U*_iso_(H) = 1.2*U*_eq_(C) for C*sp* ^3^—H, *d*(C—H) = 0.98 Å, *U*_iso_ = 1.5*U*_eq_(C) for methyl group.

## Supplementary Material

Crystal structure: contains datablock(s) I, New_Global_Publ_Block. DOI: 10.1107/S2056989017006867/hb7674sup1.cif

Structure factors: contains datablock(s) I. DOI: 10.1107/S2056989017006867/hb7674Isup2.hkl

Supporting information file. DOI: 10.1107/S2056989017006867/hb7674Isup3.cml

CCDC reference: 1548456

Additional supporting information:  crystallographic information; 3D view; checkCIF report

## Figures and Tables

**Table 1:** Hydrogen-bond geometry (Å, °)

*D*—H⋯*A*	*D*—H	H⋯*A*	*D*⋯*A*	*D*—H⋯*A*
C7—H7*B*⋯O2^i^	0.99	2.57	3.3929 (14)	141

Symmetry code: (i) 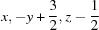.

**Table 2:** Experimental and calculated bond lengths (Å)

Bond	X-ray	B3LYP (6–311G*)
O1—C1	1.2059 (13)	1.2069
O2—C4	1.2034 (13)	1.2069
N1—C1	1.3924 (14)	1.4019
N1—C4	1.3915 (14)	1.4011
N1—C6	1.4590 (13)	1.4607
C1—C2	1.4933 (14)	1.4972
C2—C3	1.3911 (15)	1.3968
C3—C4	1.4984 (14)	1.4971
C2—C5	1.3869 (14)	1.3896
C6—C7	1.5235 (15)	1.5318
C7—C8	1.5225 (15)	1.5317
C8—C9	1.5238 (16)	1.5323
C9—C10	1.5221 (15)	1.5322
C10—C11	1.5230 (16)	1.5322
C11—C12	1.5223 (16)	1.5323
C12—C13	1.5235 (17)	1.5321
C13—C14	1.5200 (17)	1.5324
C14—C15	1.5236 (19)	1.5303

**Table 3:** Experimental details

Crystal data	
Chemical formula	C_30_H_44_N_2_O_4_
*M*_r_	496.67
Crystal system, space group	Monoclinic, *P*2_1_/*c*
Temperature (K)	173
*a*, *b*, *c* (Å)	30.6365 (16), 5.0149 (3), 8.9393 (5)
β (°)	93.980 (3)
*V* (Å^3^)	1370.11 (13)
*Z*	2
Radiation type	Mo *K*α
μ (mm^−1^)	0.08
Crystal size (mm)	0.66 × 0.65 × 0.13

Data collection	
Diffractometer	Bruker APEXII CCD
Absorption correction	Multi-scan (*SADABS*; Bruker, 2014 ▸)
*T*_min_, *T*_max_	0.703, 0.746
No. of measured, independent and observed [*I* > 2σ(*I*)] reflections	22193, 3364, 2964
*R*_int_	0.026
(sin θ/λ)_max_ (Å^−1^)	0.667

Refinement	
*R*[*F* ^2^ > 2σ(*F* ^2^)], *wR*(*F* ^2^), *S*	0.041, 0.113, 1.09
No. of reflections	3364
No. of parameters	164
H-atom treatment	H-atom parameters constrained
Δρ_max_, Δρ_min_ (e Å^−3^)	0.33, −0.20

Computer programs: *APEX2* and *SAINT* (Bruker, 2014 ▸), *SHELXS97* and *SHELXTL* (Sheldrick, 2008 ▸), *SHELXL2014* (Sheldrick, 2015 ▸), *DIAMOND* (Brandenburg, 2010 ▸) and *publCIF* (Westrip, 2010 ▸).

## supplementary crystallographic information

### Crystal data

**Table:**

C_30_H_44_N_2_O_4_	*F*(000) = 540
*M**_r_* = 496.67	*D*_x_ = 1.204 Mg m^−^^3^
Monoclinic, *P*2_1_/*c*	Mo *K*α radiation, λ = 0.71073 Å
*a* = 30.6365 (16) Å	Cell parameters from 9966 reflections
*b* = 5.0149 (3) Å	θ = 3.3–28.1°
*c* = 8.9393 (5) Å	µ = 0.08 mm^−^^1^
β = 93.980 (3)°	*T* = 173 K
*V* = 1370.11 (13) Å^3^	Block, colourless
*Z* = 2	0.66 × 0.65 × 0.13 mm

### Data collection

**Table:**

Bruker APEXII CCD diffractometer	2964 reflections with *I* > 2σ(*I*)
φ and ω scans	*R*_int_ = 0.026
Absorption correction: multi-scan (SADABS; Bruker, 2014)	θ_max_ = 28.3°, θ_min_ = 1.3°
*T*_min_ = 0.703, *T*_max_ = 0.746	*h* = −40→40
22193 measured reflections	*k* = −6→6
3364 independent reflections	*l* = −11→10

### Refinement

**Table:**

Refinement on *F*^2^	Primary atom site location: structure-invariant direct methods
Least-squares matrix: full	Hydrogen site location: inferred from neighbouring sites
*R*[*F*^2^ > 2σ(*F*^2^)] = 0.041	H-atom parameters constrained
*wR*(*F*^2^) = 0.113	*w* = 1/[σ^2^(*F*_o_^2^) + (0.0502*P*)^2^ + 0.4968*P*] where *P* = (*F*_o_^2^ + 2*F*_c_^2^)/3
*S* = 1.09	(Δ/σ)_max_ = 0.001
3364 reflections	Δρ_max_ = 0.33 e Å^−^^3^
164 parameters	Δρ_min_ = −0.20 e Å^−^^3^
0 restraints	

### Special details

**Table:**

Geometry. All esds (except the esd in the dihedral angle between two l.s. planes) are estimated using the full covariance matrix. The cell esds are taken into account individually in the estimation of esds in distances, angles and torsion angles; correlations between esds in cell parameters are only used when they are defined by crystal symmetry. An approximate (isotropic) treatment of cell esds is used for estimating esds involving l.s. planes.

### Fractional atomic coordinates and isotropic or equivalent isotropic displacement parameters (Å^2^)

**Table:**

	*x*	*y*	*z*	*U*_iso_*/*U*_eq_	
C3	0.46219 (3)	0.6062 (2)	1.04594 (11)	0.0174 (2)	
O1	0.40031 (3)	0.12036 (16)	0.84589 (9)	0.0248 (2)	
O2	0.40323 (3)	0.85647 (16)	1.14895 (9)	0.02379 (19)	
N1	0.39028 (3)	0.47982 (19)	1.00209 (10)	0.0198 (2)	
C2	0.46141 (3)	0.3850 (2)	0.95188 (11)	0.0175 (2)	
C4	0.41601 (3)	0.6734 (2)	1.07673 (11)	0.0185 (2)	
C5	0.49907 (3)	0.2709 (2)	0.90256 (11)	0.0183 (2)	
H5	0.4984	0.1198	0.8383	0.022*	
C1	0.41477 (3)	0.3030 (2)	0.92185 (12)	0.0189 (2)	
C6	0.34303 (3)	0.4510 (2)	1.00787 (13)	0.0233 (2)	
H6A	0.3336	0.5442	1.0977	0.028*	
H6B	0.3358	0.2597	1.0174	0.028*	
C7	0.31819 (4)	0.5643 (3)	0.86860 (13)	0.0251 (2)	
H7A	0.3175	0.7612	0.8764	0.030*	
H7B	0.3340	0.5180	0.7793	0.030*	
C8	0.27152 (4)	0.4597 (3)	0.84738 (13)	0.0257 (3)	
H8A	0.2554	0.5098	0.9355	0.031*	
H8B	0.2722	0.2626	0.8417	0.031*	
C9	0.24734 (4)	0.5696 (3)	0.70588 (14)	0.0275 (3)	
H9A	0.2444	0.7651	0.7165	0.033*	
H9B	0.2652	0.5359	0.6197	0.033*	
C10	0.20209 (4)	0.4500 (3)	0.67147 (14)	0.0288 (3)	
H10A	0.1840	0.4845	0.7570	0.035*	
H10B	0.2049	0.2544	0.6607	0.035*	
C12	0.13370 (4)	0.4426 (3)	0.49163 (14)	0.0305 (3)	
H12A	0.1152	0.4744	0.5765	0.037*	
H12B	0.1367	0.2474	0.4797	0.037*	
C11	0.17877 (4)	0.5626 (3)	0.52925 (14)	0.0292 (3)	
H11A	0.1757	0.7579	0.5408	0.035*	
H11B	0.1972	0.5307	0.4442	0.035*	
C13	0.11075 (4)	0.5574 (3)	0.34923 (14)	0.0312 (3)	
H13A	0.1079	0.7527	0.3611	0.037*	
H13B	0.1292	0.5249	0.2644	0.037*	
C14	0.06564 (4)	0.4394 (3)	0.31123 (16)	0.0378 (3)	
H14A	0.0469	0.4755	0.3948	0.045*	
H14B	0.0683	0.2436	0.3012	0.045*	
C15	0.04360 (5)	0.5520 (4)	0.16677 (17)	0.0469 (4)	
H15A	0.0394	0.7445	0.1778	0.070*	
H15B	0.0151	0.4656	0.1460	0.070*	
H15C	0.0621	0.5182	0.0836	0.070*	

### Atomic displacement parameters (Å^2^)

**Table:**

	*U*^11^	*U*^22^	*U*^33^	*U*^12^	*U*^13^	*U*^23^
C3	0.0191 (5)	0.0168 (5)	0.0161 (5)	0.0019 (4)	0.0005 (4)	0.0016 (4)
O1	0.0268 (4)	0.0240 (4)	0.0230 (4)	−0.0051 (3)	−0.0021 (3)	−0.0033 (3)
O2	0.0258 (4)	0.0219 (4)	0.0241 (4)	0.0038 (3)	0.0050 (3)	−0.0017 (3)
N1	0.0168 (4)	0.0238 (5)	0.0186 (4)	−0.0001 (3)	−0.0004 (3)	0.0000 (4)
C2	0.0200 (5)	0.0165 (5)	0.0157 (5)	−0.0005 (4)	−0.0013 (4)	0.0016 (4)
C4	0.0201 (5)	0.0193 (5)	0.0162 (5)	0.0011 (4)	0.0006 (4)	0.0035 (4)
C5	0.0218 (5)	0.0159 (5)	0.0170 (5)	0.0008 (4)	−0.0002 (4)	−0.0010 (4)
C1	0.0208 (5)	0.0191 (5)	0.0164 (5)	0.0000 (4)	−0.0009 (4)	0.0031 (4)
C6	0.0156 (5)	0.0321 (6)	0.0222 (5)	−0.0020 (4)	0.0008 (4)	0.0023 (4)
C7	0.0184 (5)	0.0313 (6)	0.0252 (6)	−0.0014 (4)	−0.0017 (4)	0.0041 (5)
C8	0.0186 (5)	0.0324 (6)	0.0257 (6)	−0.0017 (4)	−0.0012 (4)	0.0019 (5)
C9	0.0201 (5)	0.0341 (6)	0.0279 (6)	−0.0006 (5)	−0.0026 (4)	0.0019 (5)
C10	0.0215 (6)	0.0345 (7)	0.0296 (6)	−0.0016 (5)	−0.0040 (5)	0.0014 (5)
C12	0.0229 (6)	0.0371 (7)	0.0305 (6)	−0.0020 (5)	−0.0055 (5)	0.0009 (5)
C11	0.0218 (5)	0.0355 (7)	0.0296 (6)	−0.0009 (5)	−0.0043 (5)	0.0017 (5)
C13	0.0241 (6)	0.0388 (7)	0.0298 (6)	−0.0005 (5)	−0.0049 (5)	0.0011 (5)
C14	0.0253 (6)	0.0519 (9)	0.0349 (7)	−0.0032 (6)	−0.0074 (5)	0.0005 (6)
C15	0.0311 (7)	0.0702 (11)	0.0374 (8)	0.0019 (7)	−0.0111 (6)	0.0015 (7)

### Geometric parameters (Å, º)

**Table:**

C3—C5^i^	1.3873 (14)	C9—C10	1.5221 (15)
C3—C2	1.3911 (15)	C9—H9A	0.9900
C3—C4	1.4984 (14)	C9—H9B	0.9900
O1—C1	1.2059 (13)	C10—C11	1.5230 (16)
O2—C4	1.2034 (13)	C10—H10A	0.9900
N1—C4	1.3915 (14)	C10—H10B	0.9900
N1—C1	1.3924 (14)	C12—C11	1.5223 (16)
N1—C6	1.4590 (13)	C12—C13	1.5235 (17)
C2—C5	1.3869 (14)	C12—H12A	0.9900
C2—C1	1.4933 (14)	C12—H12B	0.9900
C5—C3^i^	1.3872 (14)	C11—H11A	0.9900
C5—H5	0.9500	C11—H11B	0.9900
C6—C7	1.5235 (15)	C13—C14	1.5200 (17)
C6—H6A	0.9900	C13—H13A	0.9900
C6—H6B	0.9900	C13—H13B	0.9900
C7—C8	1.5225 (15)	C14—C15	1.5236 (19)
C7—H7A	0.9900	C14—H14A	0.9900
C7—H7B	0.9900	C14—H14B	0.9900
C8—C9	1.5238 (16)	C15—H15A	0.9800
C8—H8A	0.9900	C15—H15B	0.9800
C8—H8B	0.9900	C15—H15C	0.9800
			
C5^i^—C3—C2	122.23 (10)	C10—C9—H9B	108.7
C5^i^—C3—C4	129.49 (10)	C8—C9—H9B	108.7
C2—C3—C4	108.27 (9)	H9A—C9—H9B	107.6
C4—N1—C1	112.56 (9)	C9—C10—C11	113.00 (11)
C4—N1—C6	125.64 (9)	C9—C10—H10A	109.0
C1—N1—C6	121.77 (9)	C11—C10—H10A	109.0
C5—C2—C3	122.78 (10)	C9—C10—H10B	109.0
C5—C2—C1	129.49 (10)	C11—C10—H10B	109.0
C3—C2—C1	107.72 (9)	H10A—C10—H10B	107.8
O2—C4—N1	126.50 (10)	C11—C12—C13	113.21 (11)
O2—C4—C3	128.08 (10)	C11—C12—H12A	108.9
N1—C4—C3	105.42 (9)	C13—C12—H12A	108.9
C2—C5—C3^i^	114.99 (10)	C11—C12—H12B	108.9
C2—C5—H5	122.5	C13—C12—H12B	108.9
C3^i^—C5—H5	122.5	H12A—C12—H12B	107.7
O1—C1—N1	125.71 (10)	C12—C11—C10	113.81 (11)
O1—C1—C2	128.31 (10)	C12—C11—H11A	108.8
N1—C1—C2	105.97 (9)	C10—C11—H11A	108.8
N1—C6—C7	112.01 (9)	C12—C11—H11B	108.8
N1—C6—H6A	109.2	C10—C11—H11B	108.8
C7—C6—H6A	109.2	H11A—C11—H11B	107.7
N1—C6—H6B	109.2	C14—C13—C12	113.54 (11)
C7—C6—H6B	109.2	C14—C13—H13A	108.9
H6A—C6—H6B	107.9	C12—C13—H13A	108.9
C8—C7—C6	112.63 (10)	C14—C13—H13B	108.9
C8—C7—H7A	109.1	C12—C13—H13B	108.9
C6—C7—H7A	109.1	H13A—C13—H13B	107.7
C8—C7—H7B	109.1	C13—C14—C15	112.82 (13)
C6—C7—H7B	109.1	C13—C14—H14A	109.0
H7A—C7—H7B	107.8	C15—C14—H14A	109.0
C7—C8—C9	112.15 (10)	C13—C14—H14B	109.0
C7—C8—H8A	109.2	C15—C14—H14B	109.0
C9—C8—H8A	109.2	H14A—C14—H14B	107.8
C7—C8—H8B	109.2	C14—C15—H15A	109.5
C9—C8—H8B	109.2	C14—C15—H15B	109.5
H8A—C8—H8B	107.9	H15A—C15—H15B	109.5
C10—C9—C8	114.13 (10)	C14—C15—H15C	109.5
C10—C9—H9A	108.7	H15A—C15—H15C	109.5
C8—C9—H9A	108.7	H15B—C15—H15C	109.5
			
C5^i^—C3—C2—C5	0.00 (18)	C4—N1—C1—C2	2.09 (12)
C4—C3—C2—C5	−179.64 (9)	C6—N1—C1—C2	−175.94 (9)
C5^i^—C3—C2—C1	178.88 (9)	C5—C2—C1—O1	−1.11 (19)
C4—C3—C2—C1	−0.76 (11)	C3—C2—C1—O1	−179.89 (11)
C1—N1—C4—O2	176.76 (10)	C5—C2—C1—N1	178.05 (10)
C6—N1—C4—O2	−5.30 (17)	C3—C2—C1—N1	−0.73 (11)
C1—N1—C4—C3	−2.53 (11)	C4—N1—C6—C7	101.29 (12)
C6—N1—C4—C3	175.41 (9)	C1—N1—C6—C7	−80.95 (13)
C5^i^—C3—C4—O2	3.09 (18)	N1—C6—C7—C8	161.36 (10)
C2—C3—C4—O2	−177.30 (11)	C6—C7—C8—C9	−178.71 (10)
C5^i^—C3—C4—N1	−177.63 (10)	C7—C8—C9—C10	174.31 (11)
C2—C3—C4—N1	1.98 (11)	C8—C9—C10—C11	−179.78 (11)
C3—C2—C5—C3^i^	0.00 (17)	C13—C12—C11—C10	179.93 (11)
C1—C2—C5—C3^i^	−178.62 (10)	C9—C10—C11—C12	179.16 (11)
C4—N1—C1—O1	−178.71 (10)	C11—C12—C13—C14	−179.76 (12)
C6—N1—C1—O1	3.26 (17)	C12—C13—C14—C15	−178.76 (13)

Symmetry code: (i) −*x*+1, −*y*+1, −*z*+2.

### Hydrogen-bond geometry (Å, º)

**Table:**

*D*—H···*A*	*D*—H	H···*A*	*D*···*A*	*D*—H···*A*
C7—H7*B*···O2^ii^	0.99	2.57	3.3929 (14)	141

Symmetry code: (ii) *x*, −*y*+3/2, *z*−1/2.
